# Findings from a Rapid Assessment of Avoidable Blindness (RAAB) in Southern Malawi

**DOI:** 10.1371/journal.pone.0019226

**Published:** 2011-04-25

**Authors:** Khumbo Kalua, Robert Lindfield, Maxwell Mtupanyama, Davie Mtumodzi, Vincent Msiska

**Affiliations:** 1 Department of Ophthalmology, College of Medicine, University of Malawi, Blantyre, Malawi; 2 International Centre for Eye Health, London School of Hygiene and Tropical Medicine, London, United Kingdom; 3 Ministry of Health, Chiradzulu District Hospital, Chiradzulu, Malawi; 4 Ministry of Health, Thyolo District Hospital, Thyolo, Malawi; 5 Ministry of Health, Mzuzu Central Hospital, Mzuzu, Malawi; Finnish Institute of Occupational Health, Finland

## Abstract

**Background:**

Data on prevalence and causes of avoidable blindness in Malawi are not readily available. The purpose of this study was to determine the prevalence and causes of blindness in persons aged 50 and above in southern Malawi to plan eye care services for the community.

**Methodology:**

A population-based survey was conducted in 7 districts in southern Malawi. Villages were selected by probability proportionate to size within each district. Clusters were further subdivided into segments. A predetermined number of segments were selected randomly in each cluster. The survey team moved from house to house in each segment until they had examined 50 people over the age of 50. Examination consisted of visual acuity measurement with tumbling “E” chart and ocular examination by an ophthalmologist. Participants were categorized by visual acuity. Those who were visually impaired (VA<6/18 in the better eye with available correction) were assigned a main cause of visual loss. Further information was sought from anyone who had received cataract surgery.

**Results:**

A total number of 3,583 persons aged 50 and above were sampled; among these 3,430 (95.7%) were examined. The prevalence of blindness (presenting visual acuity <3/60 in the better eye) among persons aged 50 and above was 3.3% (95% CI 2.5–4.1). Cataract was the most common cause of blindness contributing to 48.2% of all cases, followed by glaucoma (15.8%) and cornea scarring (12.3%). The cataract surgical coverage in blind persons was 44.6%.

**Conclusion:**

The prevalence of blindness and visual impairment in persons aged 50 and above was lower than the WHO estimate for Malawi. The majority of the causes were avoidable, with cataract accounting for approximately half of all cases of blindness. The data suggests that expansion of eye care programs to address avoidable causes of blindness is necessary in this area of southern Malawi.

## Introduction

Vision 2020: The Right to Sight, is a global initiative to eliminate avoidable blindness by the year 2020 [1], [2], [3]. It is a collaboration between the International Agency for the Prevention of Blindness (an organization representing non-governmental bodies working in prevention of blindness) and the World Health Organization (WHO) [4], [5]. Recent WHO estimates indicate that, globally, there are 45 million blind people and 153 million with visual impairment, with the highest prevalence of blindness reported in Sub-Saharan Africa [6], [7]. Extrapolation from WHO data suggests that 1% of people (all ages) in Malawi are blind, and that 80% of these are aged 50 and above [7]. Findings from prevalence surveys in east Africa (Rwanda [8], Kenya [9] and Tanzania [10]) and other African countries [11] have revealed that the overall prevalence of blindness in these countries may be much lower than the WHO estimates. It is unclear whether this is the case in Malawi. A study conducted in the late 1990's in Southern Malawi suggested a reduction in the prevalence of blindness over the previous 16 years [12]. However this study only covered a small geographical area and no other surveys have been conducted to validate its findings. A reduction in the prevalence of blindness is supported by an increase in the number of cataract surgeries performed in Malawi from approximately 300 surgeries per million population in 2003 to the current estimate (2010) of 850 surgeries per million population per year.

As part of the strategy to achieve the aim of VISION 2020 in Malawi, the Government of Malawi, in collaboration with Sight Savers, recently launched a five year Comprehensive Eye Service (CES) in the South West Zone of Malawi which comprises of 7 districts. This required robust information to plan the delivery of effective services to the population.

The Rapid Assessment of Avoidable Blindness (RAAB) survey is a relatively cheap and easy means of generating population-based data on prevalence and causes of blindness in persons aged 50 and over. Many countries are now using the RAAB as a planning tool for Vision 2020 programmes.

The aim of this study was to conduct a RAAB survey in 7 districts within Southern Malawi, and generate information that will be used for planning a VISION 2020 programme in the area.

## Methods

The study was conducted by Ministry of Health in Malawi, with technical support from International Centre for Eye Health, London School of Hygiene and Tropical Medicine (LSHTM). Ethical clearance for the study was obtained from the National Health Services Research Committee (NHSRC) in Lilongwe and permission was sought from the district Health Administrative Officers responsible for supervision and coordination of health activities within the districts. Following explanation of the purpose of the study, written informed consent was obtained from all subjects who participated in the study.

The study took place in 7 districts from the South West Zone of Malawi (Blantyre, Chikwawa, Nsanje, Thyolo, Chiradzulu, Mwanza and Neno) with a total population of 2,141,805 (National Statistical Office results, population and housing census, Zomba, Malawi 2008) [13].

### Sample Size Calculation

A prevalence of blindness of 5% in persons aged 50 and above was used, based on experience from the trachoma surveys in that area, and other RAAB surveys in sub-Saharan Africa. Using 95% confidence interval, a precision of 20% (worst acceptable prevalence 6%), design effect (DEFF) of 1.6 and a non-response rate of 10%, a sample size of 3600 was calculated which would require 72 clusters of 50 people aged 50 and above.

### Sampling Frame

Using the National Census in 2008 as the sampling frame, a list was produced of enumeration areas (called villages) and their respective population sizes of people aged 50 years and above. Villages were then selected using probability proportionate to size.

Compact segment sampling was used to select households within each village. Each village area as divided into segments containing approximately 50 people over the age of 50 and one segment was chosen at random by drawing lots. Starting at the edge of the segment, all households in the segment were included in the sample sequentially until 50 people aged 50 and above were identified. To be eligible for inclusion an individual had to reside in a household for at least six months of the previous year.

The survey was carried out by two survey teams each led by an ophthalmologist. The study took place between November 2009 and March 2010 with breaks in December and January due to heavy rain. In each cluster, the survey team visited households door-to-door, accompanied by community health workers working in the village or a local village guide (volunteer). All the examinations were conducted in the household compound.

When an eligible person was absent, the survey team left a message and returned at the end of the day to examine the individual before leaving the area. In rare circumstances where the individual was not available, information about their visual status was sought from relatives or neighbours.

### Eye Examination

Information was gathered using a standardised RAAB questionnaire and a standard protocol was followed for examination. VA was measured with a tumbling illiterate Snellen “E” chart using optotype size 6/18 on one side and size 6/60 on the other. All measurements were taken in full daylight with available spectacle correction. If the VA was less than 6/18 in either eye, then pinhole vision was also measured. A simple ocular examination was performed on every participant. This included an undilated assessment of the lens using a direct ophthalmoscope. If there was any uncertainty about the diagnosis then the individual was dilated for further examination. The principal cause of blindness or visual impairment was recorded according to the standard WHO convention.

### Training

Two teams made up of an ophthalmologist and ophthalmic clinical officer, received one week of training by an experienced trainer (RL) and had another week refresher training before starting the survey. In both training sessions inter-observer agreement was measured through repeat examination of 50 patients by each of the two teams. Agreement between the teams was assessed using kappa and had to be greater than 0.7 to proceed with the survey.

### Data entry and Analysis

Two data entry clerks were trained in data entry and double entered the data from the survey into the RAAB software programme which allowed assessment of data entry errors and missing data.

## Results

3,583 persons aged 50 and above were included in the sample. Among these 3,430 (95.7%) were examined. 1,361(38%) were males and 2,222 (62%) were females. Coverage, absenteeism and refusals are shown in Table 1. Overall 3.6% were not available, 0.1% refused and 0.5% were not capable of completing an examination. Some participants were unaware of their date of birth. The state of emergency in Malawi during 1959 was used as entry criteria for the study. If the individual was alive in 1959 then they were definitely older than 50 years. Table 2 shows age and sex structure of the population of persons aged 50 and above from the southern region of Malawi.

**Table 1:** Demography of coverage, absenteeism and refusals.

	Male		Female		Total	
	N	%	N	%	N	%
Examined	1296	95.2	2134	96	3430	95.7
Not available	58	4.3	72	3.2	130	3.6
Refused	1	0.1	4	0.2	5	0.1
Not capable	6	0.4	12	0.5	18	0.5
**Total**	**1361**	**100**	**2222**	**100**	**3583**	**100**
% of all examined	38		62		100	

**Table 2:** Age and sex structure of the population of persons aged 50 and above from the entire southern region of Malawi.

Age	Male		Female		Total
	N	%	N	%	
50–59	95964	45.1	116916	54.9	212880
60–69	63697	46.0	74860	54.0	138557
70–79	39468	42.7	53071	57.3	92539
80–89	18262	38.4	29291	61.6	47553
90+	7738	34.9	14454	65.1	22192
**Total**	**225129**	**43.8**	**288592**	**56.2**	**513721**

Table 3 describes the distribution of visual acuity with available correction in the better eye down into blindness (VA<3/60 in the better eye with available correction), severe visual impairment (SVI) (VA<6/60 to ≥3/60 in the better eye with available correction) and visual impairment (VI) (VA<6/18 to ≥6/60 in the better eye with available correction). The overall prevalence of blindness (unadjusted) from all causes in the examined adults aged 50 and above was 3.3% (95% CI 2.5–4.1). Twenty two (0.6%) people examined had bilateral aphakia/pseudophakia while 34(1%) had unilateral aphakia/pseudophakia.

**Table 3:** The distribution by visual acuity with available correction in the better eye.

Visual Acuity	Males (N = 1296)				Females (N = 2134)				Total (N = 3430)			
	N	%	95% CI		N	%	95% CI		N	%	95% CI	
Blind	47	3.6	2.5	4.8	67	3.1	2.2	4	114	3.3	2.5	4.1
Severe Visual Impairment	41	3.2	2.1	4.2	53	2.5	1.7	3.2	94	2.7	2.1	3.4
Visual Impairment	126	9.7	7.9	11.6	200	9.4	7.9	10.9	326	9.5	8.2	10.8
Bilateral aphakia/Pseudophakia	13	1	0.5	1.5	9	0.4	0.1	0.7	22	0.6	0.4	0.9
Unilateral aphakia/Pseudophakia	16	1.2	0.7	1.8	18	0.8	0.4	1.3	34	1	0.6	1.4

Table 4 describes the causes of visual loss in those examined. Untreated cataract was the commonest cause of blindness, SVI and VI contributing to 48.2% of all blind persons, 57.4% of SVI and 46.3% of VI. Glaucoma was the second most common cause of blindness (15.8%) followed by other non-trachomatous corneal scarring (12.3%).

**Table 4:** Causes of blindness, SVI and VI in persons examined.

Cause	Blind		SVI		VI	
	N	%	N	%	N	%
Cataract untreated	55	48.2	54	57.4	151	46.3
Glaucoma	18	15.8	13	13.8	12	3.7
Other cornea scar	14	12.3	2	2.1	7	2.1
Other post segment CNS	10	8.8	10	10.6	16	4.9
Phthisis	9	7.9	0	0.0	2	0.6
Trachoma	5	4.4	0	0.0	3	0.9
Surgical complication	2	1.8	2	2.1	1	0.3
Aphakia uncorrected	1	0.9	0	0.0	0	0.0
Refractive errors	0	0.0	13	13.8	134	41.1
Onchocerciasis	0	0.0	0	0.0	0	0.0
Diabetic retinopathy	0	0.0	0	0.0	0	0.0
Globe abnormality	0	0.0	0	0.0	0	0.0
AMD	0	0.0	0	0.0	0	0.0
		0.0		0.0		0.0
**Total**	**114**	100.0	**94**	100.0	**326**	100.0

Avoidable causes of blindness accounted for 75.4% of all cases. Two of the original VISION 2020 priority diseases (trachoma & onchocerciasis) had a very low prevalence, with trachoma contributing to 4.4% of all cases of blindness and onchocerciasis being completely absent. 1.8% of blindness was attributed to cataract surgical complications.

Table 5 describes the cataract surgical coverage (the number of people in the survey who had cataract surgery compared to the number who required it). The cataract surgical coverage (CSC) for blind persons was 44.6% (61.5% for males and 29.5% for females), for SVI persons it was 34.5% (51% males, 22.4% females) and for those with VI it was 15.8% (24.8% males, 10.3% females). Males had a much higher CSC at all levels of visual acuity. The overall CSC for blind eyes was low (27.0%).

**Table 5:** Cataract surgical coverage in persons and eyes.

Persons	Male	Female	Total
	%	%	%
VA<3/60	61.5	29.5	44.6
VA<6/60	51	22.4	34.5
VA<6/18	24.8	10.3	15.8
**Eyes**			
VA<3/60	36.5	20.7	27
VA<6/60	27.8	16	20.7
VA<6/18	15.8	7.8	10.5

In terms of barriers to cataract surgery, old age (‘no need felt’) was reported to be the commonest barrier (23.5%) followed by ‘no one to accompany’ (22.1%), ‘no services nearby’ (13.2) and ‘unaware that treatment was available’ (11.8%).

Table 6 shows the visual acuity in people who had undergone cataract surgery tabulated by available correction and best correction. Presenting post-operative visual acuity was good (can see 6/18) in 40.8% of persons who had IOL surgery, and improved to 56.3% after best correction. Presenting visual acuity was poor (could not see 6/60) in 32.4% of persons and, even after best correction, 28.2% still had poor visual acuity. Among the eyes with poor outcomes and which had been operated less than 3 years ago, the causes of poor outcome were attributed to pre-operative morbidity (selection) in 40%, to surgical complications in 46.7% and long term sequel in 13.3%.

**Table 6:** Visual acuity from operated eyes obtained after cataract surgery.

Correction	Visual acuity	IOL eyes (N = 71)		No IOL (N = 7)		All (N = 78)	
		N	%	N	%	N	%
Available correction	Can see 6/18	29	40.8	1	14.3	30	38.5
	Cannot see 6/18 but can see 6/60	19	26.8	1	14.3	20	25.6
	Cannot see 6/60	23	32.4	5	71.4	28	35.9
Best correction	Can see 6/18	40	56.3	1	14.3	41	52.6
	Cannot see 6/18 but can see 6/60	11	15.5	1	14.3	12	15.4
	Cannot see 6/60	20	28.2	5	71.4	25	32.1

Among the persons who had non IOL surgery (N = 7), 5 (71.4%) had poor visual outcome (could not see 6/60).

The postoperative presenting visual acuity and the satisfaction levels with results of IOL surgery are shown in Table 7. 84.8% of all persons who had surgery were either very satisfied or partially satisfied. Only 3% of persons were very unsatisfied with the results of surgery.

**Table 7:** Post operative presenting visual acuity and satisfaction with results of IOL surgery.

	Very satisfied		Partially satisfied		Indifferent		Partially unsatisfied		Very unsatisfied		Total
	N	%	N	%	N	%	N	%	N	%	
Can see 6/18	24	83	5	17.2	0		0		0		29
Cannot see 6/18 but can see 6/60	10	77	8	61.5	1	8	0		0		19
Cannot see 6/60	2	8.7	11	47.8	5	22	3	13	2	9	23
**Total**	**36**	**51**	**24**	**33.8**	**6**	**8**	**3**	**4**	**2**	**3**	**71**

### Extrapolating results to the south west zone of Malawi

Using the prevalence of blindness in persons aged 50 and above (3.3%) and the national statistical estimate (approximately 5% of the population being above the age of 50 in the south west zone) this translates to a total of 3,534 blind people of whom 1,703 (48.2%) have unoperated cataract.

## Discussion

This study was conducted in order to generate information about the magnitude and causes of blindness and to use the information for planning a VISION 2020 programme in the south west zone of Malawi.

The survey found that 3.3% (95% CI 2.5–4.1) of the population over the age of 50 were blind. The main causes of blindness were cataract, glaucoma and non-trachomatous corneal scarring. It is estimated that approximately 1,703 people in the Southern Region of Malawi are blind from cataract.

There were more females (62%) than males (38%) in the sample. Table 2 shows that this is representative of the population of Southern Malawi where the census in 2008 revealed a female predominance.

The prevalence of blindness in persons aged 50 and above found in this survey is much lower than the WHO estimate (9%) [7]. This is probably a function of the limited data available at the time the WHO estimates were developed (prior to 2004), and the on-going development of prevention of blindness activities (in particular a large increase in the numbers of cataract surgeries being performed) that have become established in east and southern Africa.

The prevalence estimate in Malawi is higher than those from other RAAB surveys within the region: Rwanda (1.8%) [8];Kenya (2%) [9]; and Tanzania (2.4%) [10]; however the confidence intervals of all estimates of the prevalence of blindness overlap with the estimate from Malawi suggesting similar findings in each survey.

In this study almost 75% of blindness was avoidable. This finding mirrors the VISION 2020 estimate of avoidable blindness of 80% [5].The overall contribution of cataract as a cause of blindness (48.2%) and glaucoma (15.8) are very similar to the recent WHO estimates of causes of blindness [7]. The finding that the majority of causes of blindness are avoidable is encouraging, especially when there are new initiatives to address blindness in the area.

Even though refractive errors were not a major cause of blindness or SVI (13.8%), they were a significant cause of VI (41.1%) suggesting that a key component of any prevention of blindness programme in this area should be the correction of refractive error.

The prevalence of blindness was higher in men than women. This pattern of blindness has not been found in other RAAB surveys in east or southern Africa. The reason for the male preponderance of blindness appears to be due to many more men than women having surgery. Men were more affected by poor outcomes after surgery leaving more of them permanently blind. This finding emphasizes the importance of ensuring high quality cataract surgery as those with poor outcomes remain permanently blind.

If the cut-off for cataract surgery is taken as 6/60 (a common cut off for cataract surgery in many low income settings) then this study suggests that there are approximately 3,360 people in the survey area who might benefit from surgery in at least one eye (termed the ‘backlog’ of cataract).

The cataract surgical coverage (CSC) in this region was much lower (44.6%) than that reported from studies from Kenya (78%) [9] and Tanzania (68.8%) [10] but similar to findings from Rwanda (47%). It is important to note that, for many years, the entire region was served by one expatriate ophthalmologist based at the tertiary hospital in Blantyre who conducted infrequent cataract camps in the districts covered by this survey. The lack of frequent screening visits and access to surgical services would explain why the CSC is low and equivalent to Rwanda which also had limited services for a long time due to conflict. Malawi currently still has a very few Ophthalmologists (nine for a population of 13.6 million); and unless plans to scale up the number of individuals conducting cataract surgeries are achieved within the next few years, it is unlikely that the CSC will markedly increase.

The cataract surgical coverage was much higher in males than females, a finding that has been repeated by other researchers in many countries. It has been reported that men have a much higher chance of accessing cataract surgery services than women [14], [15], [16], [17], [18], [19], [20] and women are less likely to report a need for sight than men [21]. Recent evidence suggest that gender disparity in accessing eye services also occur in children, with more boys accessing eye services than girls [22]. Case finding activities for cataract should prioritise women and service providers should monitor the gender mix of surgeries to ensure that women are receiving the surgery they need.

Poor outcomes of cataract surgery as seen in this survey arise from both the service providers (medical staff) and consumers (patients). Doctors often perform surgeries under suboptimal conditions in outreach camps and may lack skills to handle cases effectively when complications arise, which can result in poor visual outcomes. It can be difficult to screen patients for pre-existing eye diseases (e.g. glaucoma) when they have a dense cataract which means that post-operatively their vision might not improve despite having perfect surgery. Frequently biometry is not available which means standard intraocular lenses are inserted which might not be appropriate for the patient. Finally post operative patients from eye camps are often left without adequate medication or follow up meaning that complications, such as uveitis, are more likely to arise and lead to permanent damage. One solution is for all cataract patients to be referred to dedicated eye centres where they can be appropriately assessed and operated on under the best possible conditions.

The strengths of this study were that there was recent population census data from 2008 to derive the sample, and that accurate village-level data was available to identify those older than 50 years (although this was compounded by many individuals lack of knowledge about their age). The survey was conducted by two experienced ophthalmologists whose diagnostic agreement was found to be robust.

Limitations of the study were the small numbers of people found to be blind in the survey meaning that extrapolations and sub-group analyses had wide confidence limits. The absence of specialised diagnostic equipment meant that cases of posterior segment disease such as glaucoma and age-related macular degeneration were identified by clinical examination only which meant that results for posterior segment causes of blindness must be interpreted with care. A final limitation of the RAAB methodology is that the patient reports (satisfaction with surgery, reasons for not having surgery) are not validated and whilst useful for service planners, should be explored further if more information is required.

In conclusion this survey found a lower than expected prevalence of blindness and visual impairment in persons age 50 and above in southern Malawi, with the majority of causes of blindness and visual impairment being avoidable. The survey provides important information for planning a VISION 2020 programme for the area and can be used as baseline information for an assessment of the impact of any programme at a later time.
